# Update on the clinical trial landscape: analysis of ClinicalTrials.gov registration data, 2000–2020

**DOI:** 10.1186/s13063-022-06569-2

**Published:** 2022-10-06

**Authors:** Gillian Gresham, Jill L. Meinert, Arthur G. Gresham, Steven Piantadosi, Curtis L. Meinert

**Affiliations:** 1 Department of Medicine, Cedars-Sinai Medical Center, 700 N. San Vincente Blvd, Los Angeles, CA 90048 USA; 2 Center for Clinical Trials and Evidence Synthesis, Johns Hopkins University, Baltimore, USA; 3 Ottawa, Canada; 4 Brigham and Women’s Hospital, Harvard Medical School, Boston, MA USA

## Abstract

**Background:**

The clinical trial landscape has evolved over the last two decades, shaped by advances in therapeutics and drug development and innovation in trial design and methods. The tracking of such changes became possible with trial registration, providing the public with a window into the massive clinical research enterprise. The ClinicalTrials.gov website was launched in 2000 by the NIH National Library of Medicine and is the largest clinical trial registry worldwide. The purpose of this analysis is to describe the composition and methodologic features of clinical trials as registered on ClinicalTrials.gov and to identify trends over time.

**Methods:**

We analyzed data from the publicly available Clinical Trials Transformation Initiative Aggregate Analysis of ClinicalTrials.gov (AACT) database, focusing on trials (interventional studies) started between 1 January 2000 through 31 December 2020. Characteristics of design (e.g., phase, randomization, use of masking, number of treatment groups, sample size), eligibility criteria (age groups, gender), interventions, conditions, and funders (primary sponsor) were tabulated over time, by year trial started.

**Results:**

There were 274,043 registered interventional studies (trials) included in the analysis. Most trials were reported as randomized (65%); single site (60%); parallel-group (56%); funded by other sources (e.g., individuals, universities, and community-based organizations) (65%); and involving drug interventions (55%). Notable trends include an increase in the proportion of registered trials without FDA-defined phases (“Phase N/A”) over time, a decrease in proportion of trials that involve drugs or report treatment as a primary purpose, declining sample size and time to complete trials, and an increase in proportion of trials reporting results among completed trials. The proportion of missing registration fields has also decreased over time and more trials make protocols and other documents available. There is a current need to expand the registration fields in ClinicalTrials.gov to adapt to the evolving trial designs and reduce the number of trials categorized as “other.” Observed trends may be explained by changes in trial regulations as well as expanding and evolving trial designs, interventions, and outcome types.

**Conclusions:**

Clinical trial registration has transformed how trial information is accessed, disseminated, and used. As clinical trials evolve and regulations change, trial registries, including ClinicalTrials.gov, will continue to provide a means to access and follow trials over time, thus informing future trial design and highlighting the value of this tremendous resource.

## Introduction

The clinical trial landscape has evolved over time, shaped by advances in medicine and therapeutic development and innovation in trial design and methods. The tracking of such changes became possible with trial registration, providing the public with a window into the massive clinical research enterprise. Many clinical trial registries exist globally, established with the shared objective to address issues of reporting biases, including publication bias and selective outcome reporting, and increasing clinical trial transparency and accountability, and used by the public to access clinical trial information. The research presented herein focuses on clinical trial registry data from ClinicalTrials.gov, which is managed by the US National Institute of Health (NIH) National Library of Medicine (NLM) and is currently the largest clinical trial registry worldwide.

Two decades have passed since ClinicalTrials.gov was launched in 2000, which now includes over 400,000 registered studies (interventional and observational) across 220 countries (as of May 2022) [1]. The number of trials registered in ClinicalTrials.gov has increased over time with an uptick in registration first observed in 2005, when the International Committee of Medical Journal Editors (ICMJE) required that trials under consideration for publication must be registered prior to beginning enrollment [2]. Shortly afterwards, Congress passed the Food and Drug Administration Amendments Act of 2007 (FDAAA) expanding trial registration and reporting requirements [3–5]. Around the same time, the World Health Organization established a trial registration policy in 2006, launching the International Clinical Trials Registry Platform (ICTRP). In 2016, the FDAAA 801 Final Rule was issued further clarifying and expanding the regulatory requirements and procedures for trial registration and result reporting [6]. Another important milestone for improving our ability to analyze clinical trial registration data in the USA is the availability of The Clinical Trials Transformation Initiative (CTTI) Aggregate Analysis of ClinicalTrials.gov (AACT) [7]. The CTTI AACT is a publicly available relational cloud-based database that includes aggregated and restructured data from ClinicalTrials.gov for which content is updated daily and available for download. It includes additional tables, variables, and restructured and formatted data, which has significantly facilitated and enhanced the ability for researchers to download, analyze, and summarize ClinicalTrials.gov registration data [7].

Using the publicly available registration data from the CTTI AACT database of clinical trials, we have previously reported on characteristics and trends of trials by funding source as well as analyses of trials funded by the NIH Institutes and Centers [8–10]. While our previous analyses focused primarily on the nature of completed trials over time, the overarching objective of this review is to characterize all trials registered in ClinicalTrials.gov and started between 2000 and 2020. Specifically, we aim to describe changes in trial design features over time: trial phase, allocation, masking, interventional study model, and primary purpose. We also explore patterns in the composition of registered trials with regard to the key inclusion and exclusion criteria data elements, and the quality of trial reporting over time, including missing data elements, reporting of trial results, and availability of trial documents (e.g., protocol).

## Methods

### Data source

We conducted a cross-sectional analysis of publicly available trial registration data as structured and organized through the CTTI AACT. A static copy of the ClinicalTrials.gov database is created on the first of every month and archived on the CTTI AACT website (https://aact.ctti-clinicaltrials.org/snapshots). We downloaded the static version of the database on April 1, 2021, for the purpose of the analysis. Additional details on methods and analysis of CTTI AACT database data have been described previously. Included in the analysis were clinical trials (“interventional studies” defined as “a type of clinical study in which participants are assigned to groups that receive one or more intervention/treatment/no intervention”) registered in ClinicalTrials.gov and started between 1 January 2000 and 31 December 2020. Observational studies and expanded access studies were excluded. As this is a review of aggregate-level, publicly available data, institutional review board approval is not required.

### Outcomes of interest

Characteristics of design (e.g., phase, randomization, use of masking, number of treatment groups, sample size), eligibility criteria (age groups, gender), interventions, conditions, and funders (primary sponsor) were tabulated over time and by overall status. Overall status was grouped as completed, stopped (terminated, withdrawn, or suspended), and recruiting (not yet recruiting, active, not recruiting, or enrolling by invitation). Trials were grouped by year started in 1-year increments. Trials were categorized by year started (date of first enrollment), as trials may have been registered retrospectively, especially in earlier years (e.g., a trial that started in 2001 and registered in 2007). Thus, year trial started represented a more accurate estimate for assessing trends in trial design over time.

Trial phases were defined according to FDA phases and as included in the ClinicalTrials.gov glossary of common site terms (https://clinicaltrials.gov/ct2/about-studies/glossary) and further grouped as phase 1–2, phase 3–4, and phase not applicable (N/A), defined as trials without FDA-defined phases, such as trials of devices or behavioral interventions. Trial funders were determined based upon the “lead” agency_class from the CTTI AACT sponsor table, where organizations listed as sponsors and collaborators for a particular study include US National Institutes of Health (NIH) and other US Federal agencies (“NIH/US Fed”) (e.g., FDA, CDC, US Department of Veterans Affairs), industry, and all others (e.g., individuals, universities, and community-based organizations).

All variables were defined and categorized as included in the CTTI AACT database, which represent data retrieved directly from ClinicalTrials.gov, as well as derived variables and new variables created from information available on ClinicalTrials.gov, as well as from the National Library of Medicine (NLM) (e.g., Medical Subject Headings (MeSH) for conditions and interventions). The complete data dictionary including variable names and definitions are available at the following link: https://aact.ctti-clinicaltrials.org/data_dictionary [7].

### Statistical analysis

The analysis used all available data from trials that met eligibility criteria and were registered in the ClinicalTrials.gov registration database up to May 1, 2022, and summarized by overall status, year groups, and other variables of interest, as described above. Comparisons across year groups were made using the chi-square analysis, where applicable. Year groupings were created to align with key milestones and updates to trial registration regulations over time. Start dates were selected to account for trials that may have been registered retrospectively. The frequency of missing registration data were tabulated for each variable, but could not be included in the analysis as registration fields changed over time, and some were not required in early year groupings. All tabulations and counts were independently conducted by two reviewers (AGG and JLM) using different statistical software (PostgreSQL and SAS). Discrepancies were resolved by a third reviewer (CLM or GG).

## Results

**Fig. 1:**
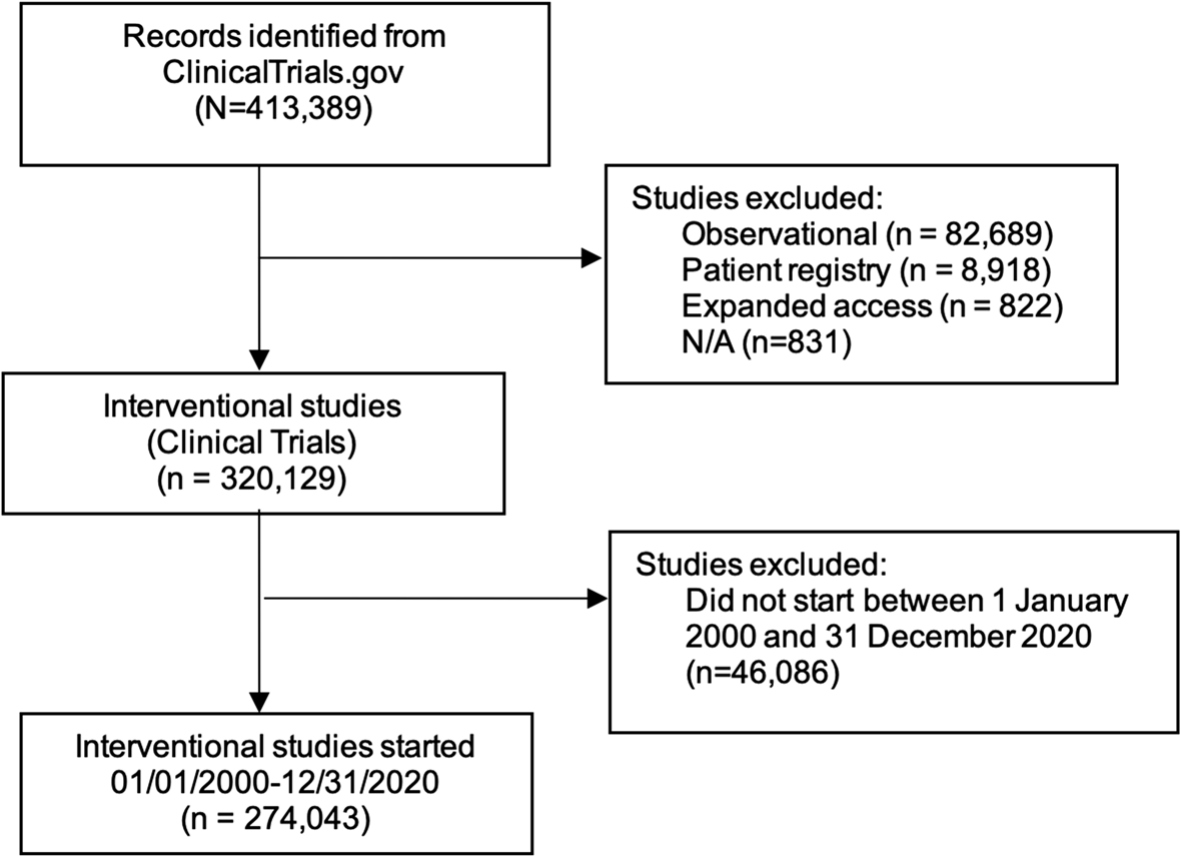
Flow chart of the included studies, as at 01 May 2022

**Fig. 2:**
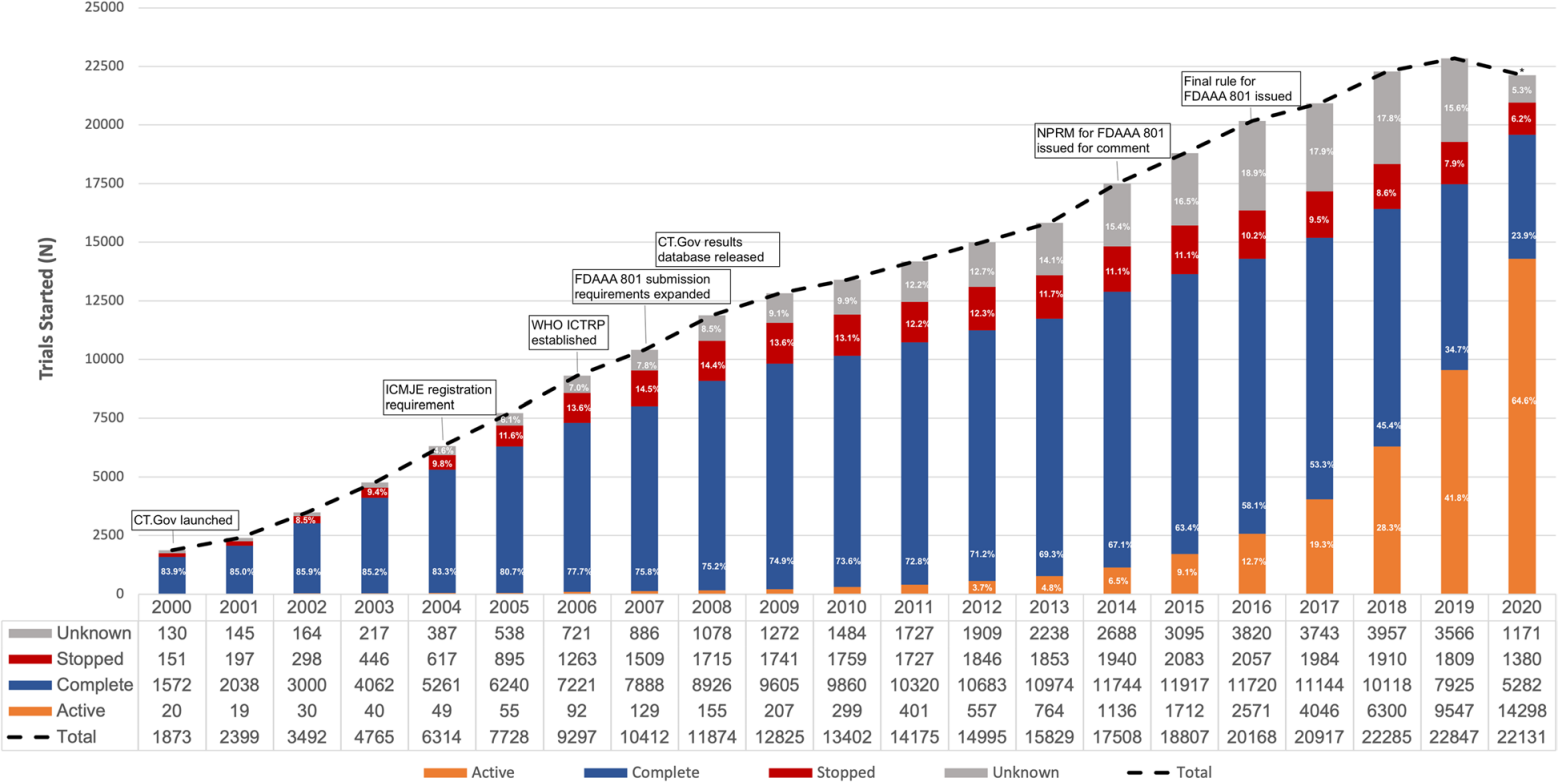
Number of trials registered in ClinicalTrials.gov, by year started and overall status

From 413,389 registered studies in ClinicalTrials.gov, as accessed on 01 May 2022, 320,129 (77%) were classified as “Interventional,” of which 274,043 had start dates between 1 January 2000 and 31 December 2020 (Fig. 1). The number of registered trials increased from 1873 trials started in 2000 to 22,131 trials started in 2020 (Fig. 2). Between 23.9 and 85.9% of registered trials were reported as complete and 6.2–14.5% of trials started were reported stopped (withdrawn, terminated, or suspended). The majority of registered trials reported to be active (open to accrual, recruiting) started between 2015 and 2020, with 64.6% open trials in 2020. A large percentage (6.9–18.9%) of registered trials have unknown status (recruitment status had not been verified in ClinicalTrials.gov for two years).

### Design characteristics

Design characteristics of registered trials started between 2000 and 2020 are displayed in Table 1. The percentage (Table 1B) of registered trials reported to be multi-site has decreased over time with 49.4% multi-site trials started in 2000, 39.3% in 2010, and 32.7% in 2020 (16.7% change since 2000). The percentage of trials reported as randomized has remained relatively stable over time (range 51.3–67.3%) with the greatest percentage of randomized trials reported in 2011. Most registered trials started between 2000 and 2020 were reported as parallel design (range 39.4–61.4%) and increased over time. Other reported intervention models, as provided and defined in ClinicalTrials.gov, include crossover trials, factorial trials, sequential design, and single group, with increases observed in reported crossover and sequential trials, and a small decrease in factorial trials. For example, since 2015, the percentage of trials reported as sequential design increased from 1% to 5.1%. The percentage of trials reported as crossover was largest between 2010 and 2015 (range 8.8–10.1%) decreasing to 6.5% in 2020 (Table 1B).

**Table 1:** Design characteristics by year started

**A. Counts of trials started by year**												
**Year started**	**Trials started**	**Multi-site**	**Allocation**	**Intervention model**					**Masked**	**Number of treatment groups (Study Arms)**		
	***N***	**>1 facility**	**Randomized**	**Single group**	**Parallel**	**Crossover**	**Factorial**	**Sequential**	**Yes**	**1**	**2**	**3+**
**2000**	1873	926	960	526	738	81	40	5	538	293	377	162
**2001**	2399	1149	1403	679	1076	143	54	3	827	398	534	243
**2002**	3492	1741	2246	1000	1746	323	64	10	1257	509	952	320
**2003**	4765	2272	3132	1442	2478	398	120	9	1877	770	1408	491
**2004**	6314	2941	4111	1951	3351	547	118	10	2504	1086	1863	661
**2005**	7728	3444	5065	2438	4153	709	165	17	3219	1412	2480	990
**2006**	9297	4194	6106	2951	5082	855	180	24	3962	1893	3528	1367
**2007**	10,412	4536	6769	3328	5799	926	165	28	4435	2446	4641	1911
**2008**	11,874	5000	7915	3669	6704	1136	195	31	5398	2906	5847	2434
**2009**	12,825	5139	8509	3941	7291	1269	180	31	5644	3194	6483	2544
**2010**	13,402	5273	8970	4012	7682	1359	230	40	5886	3309	6856	2792
**2011**	14,175	5417	9542	4119	8335	1410	207	43	6398	3426	7503	2882
**2012**	14,995	5629	10,000	4433	8784	1433	224	60	6693	3684	8104	3019
**2013**	15,829	5801	10,475	4712	9278	1472	241	83	7025	4053	8558	3137
**2014**	17,508	6580	11,587	5010	10,351	1729	251	117	7754	4265	9510	3663
**2015**	18,807	7416	12,445	5225	11,426	1663	260	187	8214	4558	10,384	3803
**2016**	20,168	7919	13,175	5500	12,376	1724	296	226	8668	4987	11,070	4044
**2017**	20,917	6511	13,663	5519	12,809	1703	265	619	9287	5151	11,523	4136
**2018**	22,285	6641	14,501	5826	13,540	1793	279	847	9848	5480	12,448	4247
**2019**	22,847	6701	14,933	6017	13,671	1894	278	987	10,163	5654	12,734	4346
**2020**	22,131	7245	14,386	5868	13,427	1443	270	1123	9672	5510	12,314	4173
**Total (/274,043)**	**274,043**	**102,475**	**179,893**	**78,166**	**160,097**	**24,010**	**4082**	**4500**	**119,269**	**64,984**	**139,117**	**51,365**
**B. Percent (%) of trials started by year**												
**Year started**	**Facilities**		**Allocation**	**Intervention model**					**Masking**	**Number of treatment groups (Study Arms)**		
	**Multi-site**		**Randomized**	**Single group**	**Parallel**	**Crossover**	**Factorial**	**Sequential**	**Yes**	**1**	**2**	**3+**
**2000**	49.4		51.3	28.1	39.4	4.3	2.1	0.3	28.7	15.6	20.1	8.6
**2001**	47.9		58.5	28.3	44.9	6.0	2.3	0.1	34.5	16.6	22.3	10.1
**2002**	49.9		64.3	28.6	50.0	9.2	1.8	0.3	36.0	14.6	27.3	9.2
**2003**	47.7		65.7	30.3	52.0	8.4	2.5	0.2	39.4	16.2	29.5	10.3
**2004**	46.6		65.1	30.9	53.1	8.7	1.9	0.2	39.7	17.2	29.5	10.5
**2005**	44.6		65.5	31.5	53.7	9.2	2.1	0.2	41.7	18.3	32.1	12.8
**2006**	45.1		65.7	31.7	54.7	9.2	1.9	0.3	42.6	20.4	37.9	14.7
**2007**	43.6		65.0	32.0	55.7	8.9	1.6	0.3	42.6	23.5	44.6	18.4
**2008**	42.1		66.7	30.9	56.5	9.6	1.6	0.3	45.5	24.5	49.2	20.5
**2009**	40.1		66.3	30.7	56.8	9.9	1.4	0.2	44.0	24.9	50.5	19.8
**2010**	39.3		66.9	29.9	57.3	10.1	1.7	0.3	43.9	24.7	51.2	20.8
**2011**	38.2		67.3	29.1	58.8	9.9	1.5	0.3	45.1	24.2	52.9	20.3
**2012**	37.5		66.7	29.6	58.6	9.6	1.5	0.4	44.6	24.6	54.0	20.1
**2013**	36.6		66.2	29.8	58.6	9.3	1.5	0.5	44.4	25.6	54.1	19.8
**2014**	37.6		66.2	28.6	59.1	9.9	1.4	0.7	44.3	24.4	54.3	20.9
**2015**	39.4		66.2	27.8	60.8	8.8	1.4	1.0	43.7	24.2	55.2	20.2
**2016**	39.3		65.3	27.3	61.4	8.5	1.5	1.1	43.0	24.7	54.9	20.1
**2017**	31.1		65.3	26.4	61.2	8.1	1.3	3.0	44.4	24.6	55.1	19.8
**2018**	29.8		65.1	26.1	60.8	8.0	1.3	3.8	44.2	24.6	55.9	19.1
**2019**	29.3		65.4	26.3	59.8	8.3	1.2	4.3	44.5	24.7	55.7	19.0
**2020**	32.7		65.0	26.5	60.7	6.5	1.2	5.1	43.7	24.9	55.6	18.9
**Average**	40.4		64.8	29.1	55.9	8.6	1.6	1.1	41.9	22.0	44.8	16.8

The percentage of registered trials reported as single or double+ masked (blinded) has been stable over time, with over 40% of trials reported as masked since 2005. Approximately one quarter of registered trials are reported to have a single treatment group (arm), while the remainder have two or more treatment groups. The percentage of trials reported to have two groups has increased with 20.1% in 2000, 32.1% in 2005, 51.2% in 2010, and 55.2% and 55.6% in 2015 and 2020, respectively (Table 1).

**Fig. 3:**
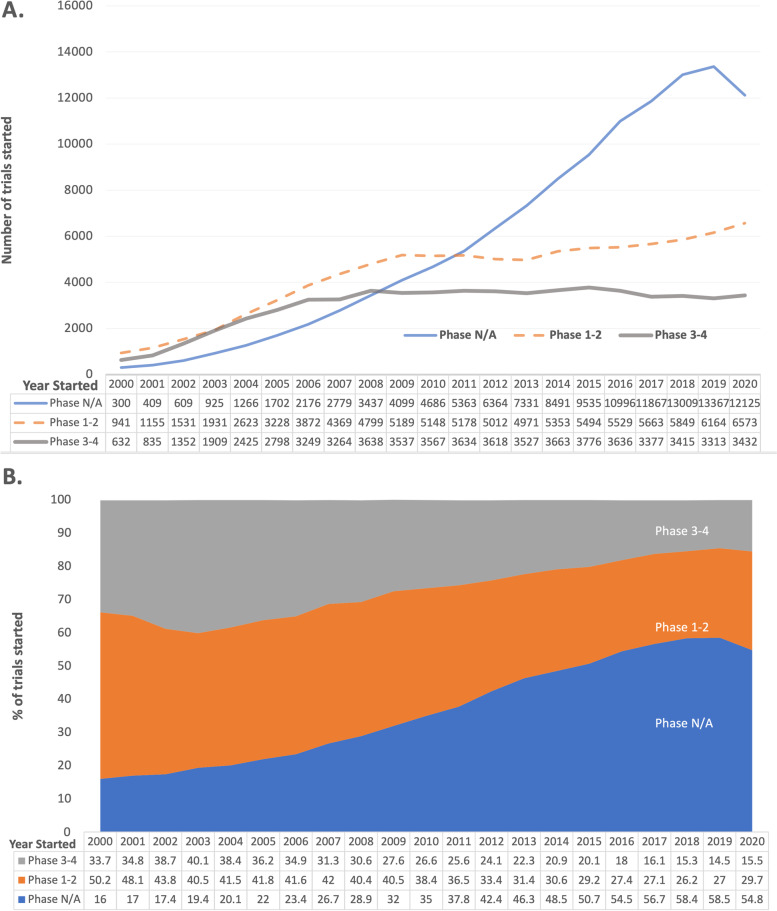
Number (**A**) and percent (**B**) of trials registered in ClinicalTrials.gov started, by year started and phase category. *there were 9 registered trials that did not report phase

Figure 3 shows the number of registered trials started by year and phase: phase N/A (non-FDA-defined phase), phase 1–2, and phase 3–4. The number of registered trials reported as “phase N/A” has increased from 300 registered trials started in 2000 to 13,367 started in 2019; this number decreased to 12,125 trials started in 2020 (Fig. 3A). The percentage of trials reported as “phase N/A” also increased from 16 to 54.8% over the past two decades. In contrast, phase 1–2 trials and phase 3–4 trials have decreased over time (Fig. 3B).

### Trial conduct and recruitment information

Trial descriptive information and recruitment details, as reported in ClinicalTrials.gov, are summarized in Table 2, including trial sponsor, presence of data safety, and monitoring committee (DSMC), availability of trial protocol, and eligibility. The majority of trials started between 2000 and 2020 report primary sponsor as “other” (e.g., individuals, universities, and community-based organizations). The relative proportion of trials reporting “other” as primary sponsor has increased over time while the proportion of trials reporting industry or NIH/other US Gov as the primary sponsor has decreased (Table 2). Trials reported having a data safety monitoring committee (DSMC) ranged from approximately 20% in 2000 to 38% in 2010 and 34.2% in 2020. The composition of trials has remained relatively stable over time, with the majority of trials involving adults and children (74.5%) and both men and women (>80%). The average percentage of trials, 2000 through 2020, conducted in women only or men only were 9.9% and 5.1%, respectively. The percentage of registered trials across all age categories (adults only, children only, or adults and children) remained relatively stable over time involving populations of all ages (adults and children) ranging from 72–81%, 16-20% among adults only, and 5.2-6.5% among (Table 2).

**Table 2:** Trial descriptive and recruitment information by year started

**A: Counts (*n*) by year started**												
**Year started**	**Trials started**	**Sponsor**			**Has DSMC**	**Protocol included**	**Sex**			**Age**		
	***N***	**Industry**	**NIH/US Gov**	**Other**	**Yes**	**Yes**	**Men only**	**Women only**	**All**	**Adults only**	**Children only**	**Adults and children**
**2000**	1873	456	323	1094	373	6	87	224	1552	248	113	1512
**2001**	2399	746	359	1294	432	3	125	286	1974	363	129	1907
**2002**	3492	1336	427	1729	683	11	173	376	2935	558	217	2717
**2003**	4765	1773	420	2572	1040	15	254	490	4012	817	292	3656
**2004**	6314	2380	549	3385	1433	36	322	651	5331	1016	380	4918
**2005**	7728	2832	499	4397	1973	55	388	707	6624	1259	489	5980
**2006**	9297	3558	523	5216	2748	67	474	863	7950	1630	581	7086
**2007**	10,412	3940	415	6057	3714	62	531	992	8883	1911	622	7879
**2008**	11,874	4364	399	7111	4239	105	652	1143	10,066	2244	689	8941
**2009**	12,825	4379	402	8044	4794	177	729	1156	10,928	2473	787	9565
**2010**	13,402	4289	459	8654	5093	211	732	1221	11,441	2619	814	9969
**2011**	14,175	4420	411	9344	5391	334	831	1304	12,024	2877	919	10,379
**2012**	14,995	4271	344	10,380	5914	554	847	1454	12,683	3069	973	10,953
**2013**	15,829	4223	352	11,254	6141	940	909	1438	13,471	3279	998	11,552
**2014**	17,508	4581	350	12,577	6545	1391	985	1684	14,822	3682	1110	12,716
**2015**	18,807	4621	378	13,808	6844	2268	967	2003	15,814	3890	1157	13,760
**2016**	20,168	4586	370	15,212	7096	3013	994	2107	17,044	4097	1317	14,754
**2017**	20,917	4529	354	16,034	7263	3310	1006	2163	17,739	4248	1338	15,331
**2018**	22,285	4875	374	17,036	7170	2814	983	2243	19,042	4416	1429	16,440
**2019**	22,847	4832	377	17,638	7370	1699	995	2241	19,602	4441	1378	17,028
**2020**	22,131	4918	270	16,943	7565	835	859	1958	19,306	4038	1159	16,934
**Total**	**274,043**	**75,909**	**8355**	**189,779**	**93,821**	**17,906**	**13,843**	**26,704**	**233,243**	**53,175**	**16,891**	**203,977**
**B: Percent (%) by year**												
	**Sponsor**			**Has DSMC**	**Protocol included**	**Eligibility: sex**^**a, b**^			**Eligibility: age (%)**			
**Year started**	**Industry**	**NIH/US Gov**	**Other**	**Yes** **(%)**	**Yes** **(%)**	**Men only (%)**	**Women only (%)**	**All (%)**	**Adults only (%)**	**Children only (%)**	**Adults and children**	
**2000**	24.3	17.2	58.4	19.9	0.3	4.6	12.0	82.9	13.2	6.0	80.7	
**2001**	31.1	15.0	53.9	18	0.1	5.2	11.9	82.3	15.1	5.4	79.5	
**2002**	38.3	12.2	49.5	19.6	0.3	5.0	10.8	84.0	16.0	6.2	77.8	
**2003**	37.2	8.8	54.0	21.8	0.3	5.3	10.3	84.2	17.1	6.1	76.7	
**2004**	37.7	8.7	53.6	22.7	0.6	5.1	10.3	84.4	16.1	6.0	77.9	
**2005**	36.6	6.5	56.9	25.5	0.7	5.0	9.1	85.7	16.3	6.3	77.4	
**2006**	38.3	5.6	56.1	29.6	0.7	5.1	9.3	85.5	17.5	6.2	76.2	
**2007**	37.8	4.0	58.2	35.7	0.6	5.1	9.5	85.3	18.4	6.0	75.7	
**2008**	36.8	3.4	59.9	35.7	0.9	5.5	9.6	84.8	18.9	5.8	75.3	
**2009**	34.1	3.1	62.7	37.4	1.4	5.7	9.0	85.2	19.3	6.1	74.6	
**2010**	32.0	3.4	64.6	38.0	1.6	5.5	9.1	85.4	19.5	6.1	74.4	
**2011**	31.2	2.9	65.9	38.0	2.4	5.9	9.2	84.8	20.3	6.5	73.2	
**2012**	28.5	2.3	69.2	39.4	3.7	5.6	9.7	84.6	20.5	6.5	73.0	
**2013**	26.7	2.2	71.1	38.8	5.9	5.7	9.1	85.1	20.7	6.3	73.0	
**2014**	26.2	2.0	71.8	37.4	7.9	5.6	9.6	84.7	21.0	6.3	72.6	
**2015**	24.6	2.0	73.4	36.4	12.1	5.1	10.7	84.1	20.7	6.2	73.2	
**2016**	22.7	1.8	75.4	35.2	14.9	4.9	10.4	84.5	20.3	6.5	73.2	
**2017**	21.7	1.7	76.7	34.7	15.8	4.8	10.3	84.8	20.3	6.4	73.3	
**2018**	21.9	1.7	76.4	32.2	12.6	4.4	10.1	85.4	19.8	6.4	73.8	
**2019**	21.1	1.7	77.2	32.3	7.4	4.4	9.8	85.8	19.4	6.0	74.5	
**2020**	22.2	1.2	76.6	34.2	3.8	3.9	8.8	87.2	18.2	5.2	76.5	
**Average**	30.0	5.1	64.8	31.5	4.5	5.1	9.9	84.8	18.5	6.1	75.4	

^a^May not add up to 100%, due to missing data (see Table 4)

^b^As defined in ClinicalTrials.gov, sex based on biological distinctions and distinct from gender-based eligibility

**Fig. 4:**
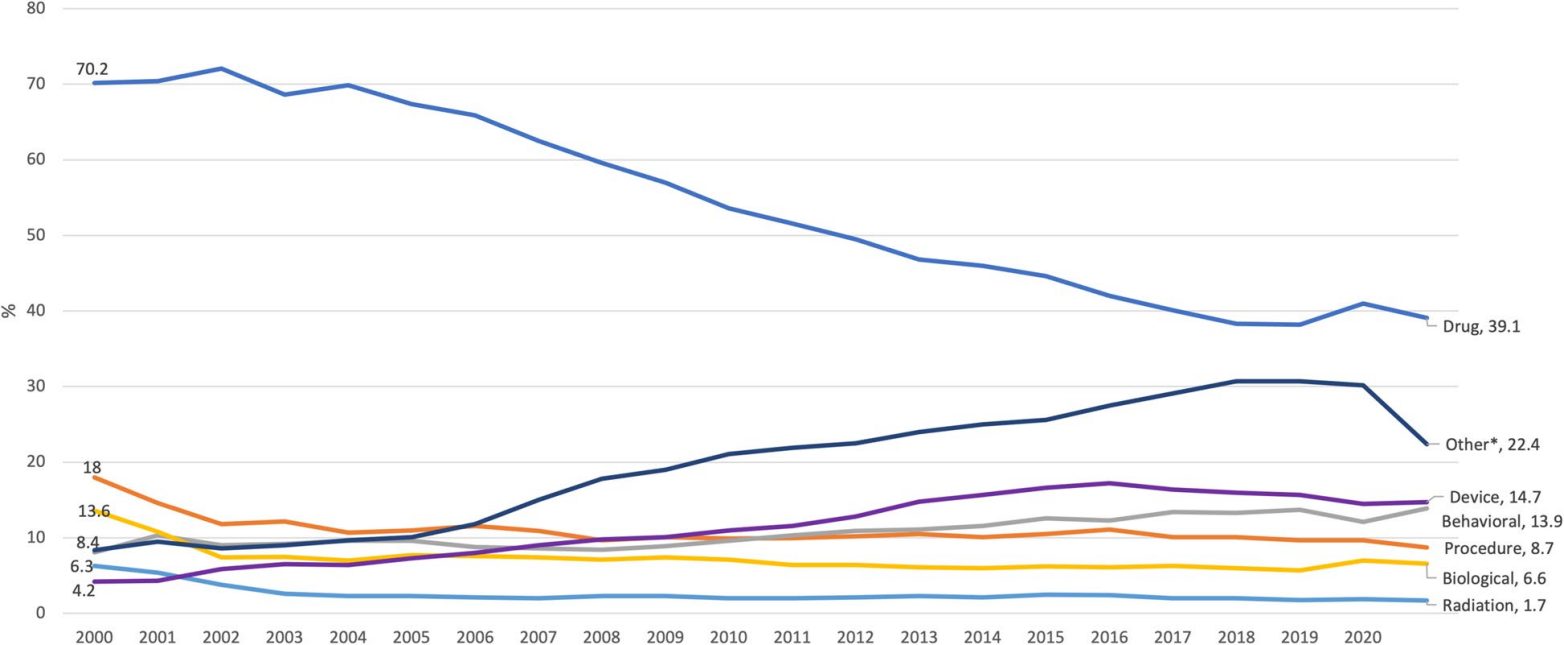
Intervention types by year started

**Fig. 5:**
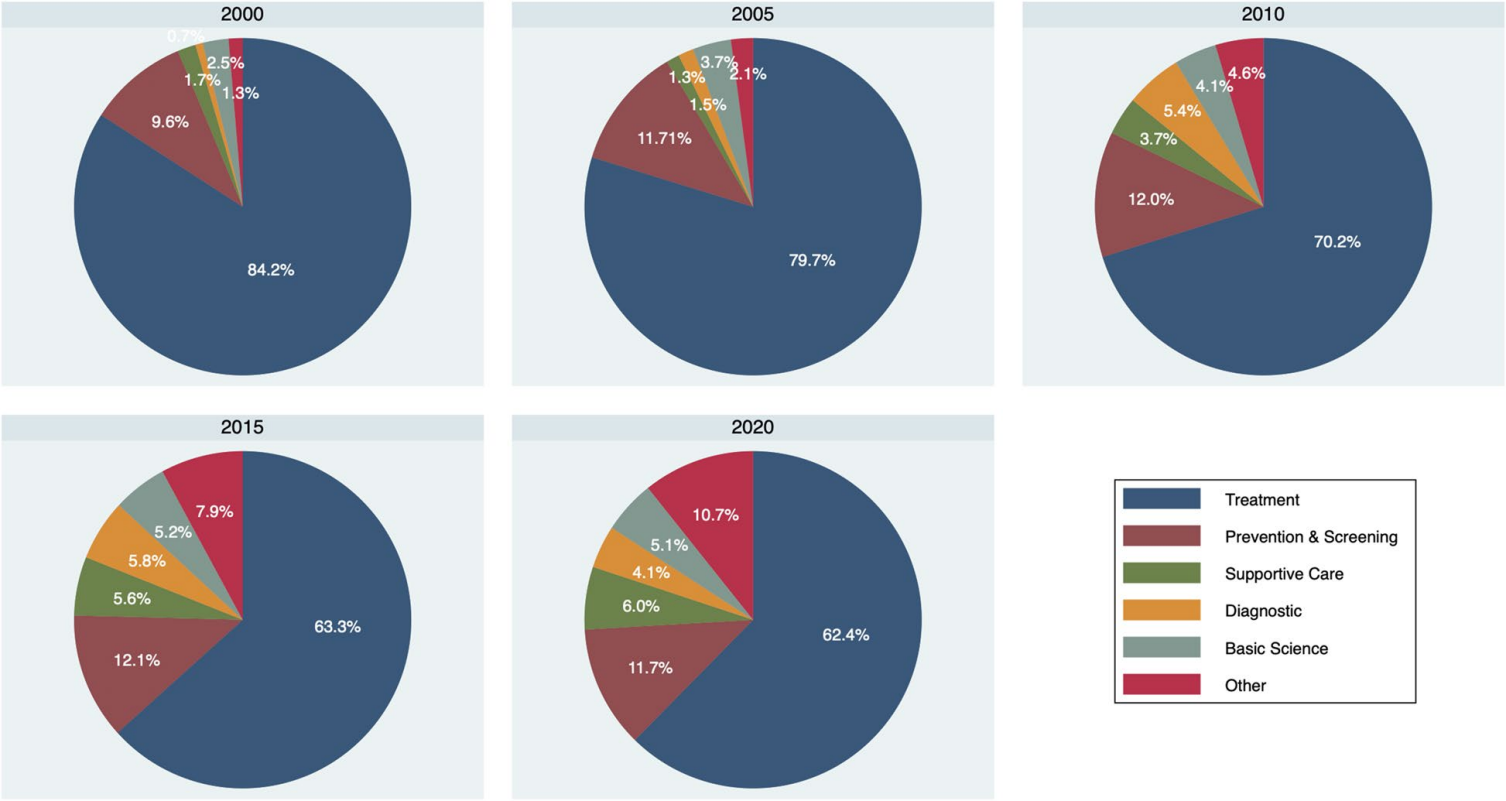
Primary purpose over time by 5-year increments

Between 2000 and 2005, the percentage of registered trials reporting “drugs” as primary intervention types decreased from 70.2% in 2000 to 39.1% in 2020. The percentage of trials involving devices, behavioral interventions, and “other intervention types” increased over time (Fig. 4). These trends are reflected in the changing percentage of trials reporting “treatment” as primary purpose over time, with 84.2% in 2000, 79.7% in 2005, 70.2% in 2010, 63.3% in 2015, and 62.4% in 2020 (Fig. 5). Of note, registered trials with primary purpose reported as “prevention and screening”, “supportive care”, and “others” increased over time (Fig. 5).

### Reporting characteristics among completed trials registered in ClinicalTrials.gov

Median trial duration has decreased over time among registered trials reported as completed in ClinicalTrials.gov. The time from the date of first enrollment to enrollment completion date, ranged from 0.6 to 4.3 years for trials starting between 2000 through 2020 (Table 3). A twofold decrease in median years to trial completion was observed between 2000 (4.3 years, IQR 2.3, 6.8) and 2007 (2.0 years, IQR 1.0, 3.7), decreasing to 1.6 years in 2015. Median trial enrollment (actual sample size) was 82 (IQR 33–256) in 2000, 69 (IQR 30, 200) in 2005, 57 (IQR 24, 149) in 2010, 60 (25, 140) in 2015, and 62 (IQR 30, 150) in 2020 (Table 3). Most completed registered trials report sample sizes <50 participants across all years, with the percentage of trials conducted in more than 500 participants decreasing over time (Table 3). The number and percentage of registered trials reporting results has increased over time, with a notable increase in 2007 (n=2840, 36%) when the ClinicalTrials.gov results database was launched, compared to previous years ranging from 8.7 to 24.5% trials with posted results. The time to report results has also improved over time, decreasing from a median of 29 months in 2007, when the result database was launched, to 12 months in 2015, and 10 months in 2020. The percentage of registered trials posting results in 12 months or less also increased after 2015, when the final rule for FDAAA 801 was issued, although remains a small (0.1–1.4%) (Table 3). This percentage reflects reporting for all completed trials, including those that do not meet the definition of an “applied clinical trial” or are required to report results. Finally, the percentage of trial registration fields with missing/null values has decreased across most required registration fields over time. Since the FDAAA 801 submission requirements were expanded in 2007, the percentage of missing responses for fields including randomization, masking, intervention model, and eligibility all decreased to <1% missing (Table 4). Registration fields including the number of facilities (sites), treatment groups, and primary purpose had a higher proportion of missing values in earlier years (2000–2015), decreasing to 0–1% by 2020.

**Table 3:** Completion status, enrollment, and result reporting among completed registered trials, 2000–2020

Year started	Completed	Time to completion	Sample size^**a**^	Sample size categories (%)					Reported results	Time to report results^**d**^	
	*N*	Years (median, IQR)	(Median, IQR)	01–50	50–100	101–500	500–1000	>1000	Yes *N* (%)	<12 mos. *N* (%)	Months Median (IQR)
**2000**	1572	4.3 (2.3, 6.8)	82 (33, 256)	38.3	17.6	28.8	8.3	7	137 (8.7)	0 (0)	49 (20, 87)
**2001**	2038	3.9 (2.0, 6.3)	91 (37, 270)	34.6	19.6	30.6	7.8	7.4	207 (10.2)	0 (0)	55 (27, 97)
**2002**	3000	3.4 (1.5, 5.5)	82 (36, 257)	37.8	18.9	31.7	7.3	4.3	359 (12.0)	0 (0)	57 (22, 84)
**2003**	4062	3.3 (1.7, 5.1)	81 (36, 251)	34.6	20.2	31.8	7.7	5.6	650 (16.0)	0 (0)	53 (23, 79)
**2004**	5261	2.9 (1.5, 4.6)	75 (33, 242)	37.7	19.0	31.9	7.8	5.2	1000 (19.0)	0 (0)	47 (21, 72)
**2005**	6240	2.6 (1.4, 4.3)	69 (30, 200)	39.2	19.2	30.8	6.3	4.5	1526 (24.5)	0 (0)	39 (19, 69)
**2006**	7221	2.3 (1.3, 3.9)	62 (29, 200)	38.4	18.9	30.7	6.8	5.2	2296 (31.8)	0 (0)	32 (14, 61)
**2007**^**b**^	7888	2.0 (1.0, 3.7)	60 (25, 168)	40.3	20.6	28.5	5.9	4.7	2840 (36.0)	0 (0)	29 (13, 56)
**2008**	8926	1.9 (0.8, 3.5)	58 (24, 150)	42.2	20.1	29.0	5.0	3.7	3165 (35.5)	9 (0.1)	27 (12, 52)
**2009**	9605	1.9 (0.9, 3.4)	58 (24, 150)	42.6	20.7	28.1	4.8	3.8	3322 (34.6)	16 (0.2)	22 (12, 44)
**2010**	9860	1.9 (0.8, 3.4)	57 (24, 149)	43.7	20.2	27.7	4.9	3.6	3136 (31.8)	20 (0.2)	19 (12, 39)
**2011**	10,320	1.8 (0.8, 3.2)	60 (25, 150)	43.1	20.7	27.6	4.8	3.8	3135 (30.4)	27 (0.3)	18 (11, 35)
**2012**	10,683	1.9 (0.9, 3.3)	59 (24, 150)	43.6	21.1	27.1	4.4	3.7	3069 (28.7)	18 (0.2)	15 (11, 30)
**2013**	10,974	1.8 (0.8, 3.2)	56 (24, 140)	45.7	21.2	25.9	4.0	3.2	3192 (29.1)	51 (0.5)	14 (11, 27)
**2014**	11,744	1.7 (0.8, 3.0)	57 (24, 140)	46.0	21.1	25.7	3.7	3.5	3107 (26.5)	51 (0.4)	13 (11, 24)
**2015**^**c**^	11,917	1.6 (0.8, 2.8)	60 (25, 140)	45.2	21.9	25.8	3.9	3.3	3034 (25.5)	57 (0.5)	12 (11, 22)
**2016**	11,720	1.5 (0.8, 2.7)	59 (24, 138)	46.4	22.0	24.5	3.6	3.5	2635 (22.5)	41 (0.3)	12 (11, 20)
**2017**	11,144	1.3 (0.7, 2.3)	60 (26, 138)	47.0	23.1	23.7	3.0	3.3	2268 (20.4)	14 (0.1)	12 (11, 18)
**2018**	10,118	1.1 (0.5, 1.9)	60 (26, 138)	49.7	22.8	22.0	2.9	2.6	1643 (16.2)	30 (0.3)	12 (10, 16)
**2019**	7925	0.8 (0.4, 1.5)	60 (28, 130)	52.7	24.0	19.0	2.1	2.2	862 (10.9)	53 (0.7)	11 (9, 13)
**2020**	5282	0.6 (0.3, 1.0)	62 (30, 150)	49.6	23.5	21.1	2.5	3.3	358 (6.8)	75 (1.4)	10 (6, 12)

^a^Actual sample size; ^b^year ClinicalTrials.gov result database launched; ^c^results required to be reported within 12 months of trial’s primary completion date for applicable clinical trials; ^d^not all results required to post results, only trials that meet definition of an applicable clinical trial

**Table 4:** Percent (%) missing responses^a^, by data element and trial start year

Year	Facilities (%)	Randomized (%)	Masked (%)	Tx groups (%)	Primary purpose (%)	Intervention model (%)	Eligibility: sex (%)
**2000**	17.4	21.5	22.5	55.6	2.6	25.8	0.5
**2001**	16.6	13.6	14.3	51.0	3.0	18.5	0.6
**2002**	17.0	7.7	6.7	49.0	5.8	10.0	0.2
**2003**	14.4	4.9	3.6	44.0	5.0	6.7	0.2
**2004**	14.0	4.0	3.4	42.8	5.0	5.3	0.2
**2005**	11.4	2.7	2.0	36.8	5.2	3.2	0.1
**2006**	9.3	1.7	1.7	27.0	4.0	2.2	0.1
**2007**	8.6	1.4	1.5	13.6	3.7	1.6	0.1
**2008**	8.3	1.2	1.0	5.8	4.3	1.2	0.1
**2009**	7.8	0.8	0.5	4.7	4.5	0.9	0.1
**2010**	7.3	0.6	0.4	3.3	4.4	0.6	0.1
**2011**	7.2	0.7	0.4	2.6	4.9	0.4	0.1
**2012**	7.9	0.5	0.4	1.3	4.2	0.4	0.1
**2013**	8.5	0.5	0.3	0.5	3.7	0.3	0.1
**2014**	10.8	0.3	0.3	0.4	3.5	0.3	0.1
**2015**	13.5	0.3	0.3	0.3	2.4	0.2	0.1
**2016**	14.9	0.3	0.4	0.3	1.1	0.2	0.1
**2017**	5.4	0.3	0.0	0.5	0.1	0.0	0.0
**2018**	4.9	0.3	0.0	0.5	0.0	0.0	0.1
**2019**	6.3	0.3	0.0	0.5	0.0	0.0	0.0
**2020**	7.6	0.1	0.0	0.6	0.0	0.0	0.0

^a^ Null or missing values for selected required data elements

## Discussion

In this study, we characterized and described trends in the design and composition of trials registered in ClinicalTrials.gov that started between 2000 through 2020. Prior to registration, there was no viable way of identifying trials except via the published literature—a biased sample since only a small fraction of trials are published. With the launch of ClinicalTrials.gov in 2000 and subsequent establishment of the World Health Organization (WHO) International Clinical Trials Registry Platform (ICTRP) in 2006, access to important trial information along with the ability to trace the state and nature of trials became possible. While it would be of interest to analyze all available registry data across multiple International Registries, differences in regulations by country and definitions, lack of a common data structure, and risk for duplicate entries make it difficult to provide an accurate account [11]. Thus, leveraging the publicly available AACT CTTI data, we provide an overview of the clinical trial landscape through the lens of ClinicalTrials.gov.

During the first 5 years from when ClinicalTrials.gov was launched, and prior to the ICMJE edict of 2005, we observed a much less complete account of trials. This is reflected in the small number of trials registered between 2000 and 2005. In the years that followed, there were important developments in trial registration regulation in the United States, including the establishment of the FDAAA section 801 in 2007, which required more trials to be registered and expansion of required data elements. Consequently, the number of trials started doubled during that year period, along with a jump in the number of trials without FDA-defined phases (phase “N/A”). The Food and Drug Administration Amendments Act of 2007 (FDAAA) also included the requirement that investigators post results of trials covered under FDA regulations on ClinicalTrials.gov within 1 year of completion. Failure to comply carries provisions for heavy fines. Although not a substitution for publication, we observed that results reporting in ClinicalTrials.gov has improved over time and represents an important step towards trial accountability and transparency.

A notable trend observed in this analysis was the decline in phase 1–4 trials and the increase in trials without FDA-defined phases, indicated as “Phase N/A” in ClinicalTrials.gov. Feasibility studies, non-drug trials, behavioral trials, and other trial designs (e.g., adaptive or platform) that do not fit within the FDA definition for phases fall into the “Phase N/A” category. Between 2016 and 2020, more than 55.9% trials were categorized “Phase N/A.” Given the broader definition and larger number of trials that do not fit within the FDA-defined phases, there is a need to update the ClinicalTrials.gov registration information capture to include additional data elements which specifically categorize more of the “N/A” study characteristics into pre-specified design classifications. When ClinicalTrials.gov was first established, emphasis was placed on FDA trials where the majority of registrations included US-funded drug trials, following the FDA definitions for the primary sponsor as the funder and holder of Investigational New Drug applications. The FDAAA “final rule” of 2016 refined the definition of an “applicable clinical trial” (ACT) and expanded on requirements for result reporting [7], supporting the need to include additional trial design options and categories in the registration elements.

As half of the trials registered in ClinicalTrials.gov are conducted outside of the USA, a third conducted in the US only, and the remainder in both US and other countries, there has been a significant increase in the number of trials funded by other sources (e.g., universities, foundations) and a smaller percentage of trials funded by the NIH/US Government or industry over time.

One improvement to the ClinicalTrials.gov registry would be to provide means to specifically identify primary funding source(s) and respective investments in each trial undertaken. Over three quarters of primary sponsors for registered trials are categorized as “other.” As trials are collaborative and often include more than one sponsor or funder, it is difficult to describe the current funding status of trials. We have previously suggested the inclusion of a funding variable and additional link or established connection to the NIH Reporter funding information for any trial funded by NIH [9, 10]. The majority of trials funded by other sources tend to be smaller, do not have FDA-defined phases, and do not have results posted, inundating the ClinicalTrials.gov registry with small, underpowered trials that are too small to answer meaningful questions [12, 13]. However, such trials are often required to generate preliminary data for grant applications and to obtain funding for larger, more informative and practice-changing trials. Thus, it would be of interest to include an additional variable in ClinicalTrials.gov to establish linkage to the subsequent larger trials, to determine how many have been funded as a result of these smaller “pilot” or feasibility trials.

Trial designs have evolved over time, and while ClinicalTrials.gov is structured to accommodate trials conducted independently and sequentially (i.e., from phase 1 to 2 to 3 to 4), there are more adaptive designs, platform trials, expansion cohorts, decentralized designs, and other methods applied to enhance trial efficiency [14, 15]. This can be observed in the increasing number of sequential designs over time, for instance, although not all trial designs are included in the drop-down menu when registering a clinical trial in ClinicalTrials.gov. Until data capture and the quality of reporting of these trial designs improve, it is difficult to know how many trials are currently being conducted [16, 17]. Additional registration fields to capture further specifics of trial design may help improve our understanding of how trial designs have changed over time, and whether the reported sample sizes are sufficient to provide meaningful answers.

Evolving designs may be driven by several factors. For one, trial outcomes have also evolved over time, with more trials using surrogate outcomes and biomarkers, composite outcomes, patient-reported outcomes, and massive lists of genomic information [18, 19]. To describe the different types of outcomes being used, an additional field specifying the outcome type or category would be informative to understand trends in trial outcomes over time. The need for additional categories and links to publications related to the primary and secondary objectives, if any, would also allow for better tracking of publications related to the registered trials. In addition, advances in technology have resulted in its integration into trial design and changed how trials are being conducted (e.g., decentralized designs) and how outcomes are being captured [20]. As technology continues to advance and becomes integrated with health care, the ClinicalTrials.gov registration fields will once again need to be reimagined. This became apparent in the year 2020, with the COVID-19 pandemic and increased use of telehealth and technology to continue study visits and assessments for many of the ongoing trials [21]. This was also marked by over 4500 additional interventional trials related to COVID-19 registered in ClinicalTrials.gov alone, as defined by the ClinicalTrials.gov “covid-19” search terms as listed on the website [22]. The impact of COVID-19 on the completion status and recruitment for non-COVID-19-related trials will continue to unfold in the years that follow, and a more in-depth analysis of the characteristics of COVID-19 trials is planned. Finally, trial designs have evolved along with the changing populations and conditions we study over time.

A limitation of this analysis is that it only includes trials registered in ClinicalTrials.gov. Although accounting for a sizable fraction of all trials, our scope is not necessarily representative of the entire clinical research enterprise. ClinicalTrials.gov is only one of many trial registries that currently exist globally. While we considered analzying all registration data as available in the WHO ICTRP, several obstacles exist to analyzing study metadata from the WHO ICTRP as a result of incomplete data, lack of a single minimum information standard for fields required, and discrepancies between fields across the WHO Trial registration datasets as noted by Miron et al. [23]. We have previously commented on the value of merging registries into a single international trial registry [10]. While the ICTRP provides a platform for multiple registries with a unique trial identifier, it only accounts for approximately 30% of registrations across 16 registries. Furthermore, trials are not registered directly through the platform, thus do not follow the same registration and reporting requirements, or share a common data structure. Therefore, trial registration platforms are at risk of including incomplete or inconsistent trial information and, for instance, duplicate registrations, without an informed standardized protocol existing to identify and merge these [23, 24]. Additionally, there remains a large number of trials that are not registered, making it difficult to obtain a complete account of all trials [25]. As observed in our analysis as well as other reports, the number of trial registrations, especially in the last decade, have increased [26].

Another limitation of this analysis is the inability to account for differences in the reporting quality and completeness of registered studies over time, due to changing policies and updates to registration elements and reporting requirements. Not all trial registration data are available over any given time frame, especially during the first 5-6 years prior to ICMJE. As observed in our analysis, however, the percentage of missing fields decreases for most required elements over time, with less than 1% missing in the later half of the decade. Although the completeness of trial reports is reviewed through the ClinicalTrials.gov Protocol Registration System (PRS), the accuracy, consistency, and quality of the data in the registry cannot be guaranteed [1, 27]. Thus, it is difficult to make accurate comparisons across time periods or data elements and these limitations should be taken into account when interpreting the findings from this analysis.

Despite its limitations, this study provides a comprehensive look at the AACT CTTI database to date, spanning over two decades and including all interventional studies registered in ClinicalTrials.gov. We summarize insights and suggestions to improve the ClinicalTrials.gov database and registration fields in order to adapt to the evolving and expanding clinical trial landscape. Future directions for this research include analyzing the ClinicalTrials.gov results database, including trial composition and demographics, primary outcome results, and safety data. Using the MeSH database, a more detailed analysis oftrials by condition, including COVID-19-related trials, will also be explored. Finally, an analysis of all registered trials across multiple clinical trials registries will be important to gain a global perspective of the International clinical trial landscape.

## Conclusion

Clinical trial registration has transformed how trial information is accessed, disseminated, and used. As clinical trials evolve and regulations change, trial registries, including ClinicalTrials.gov, will continue to provide a means to access and follow trials over time, thus informing future trial design and highlighting the value of this tremendous resource.

## Data Availability

The datasets and statistical code used for the analsis of the current study are available from the corresponding author on reasonable request.
